# Ocular infestation by a juvenile leech, Myxobdella sinanensis in Japan

**DOI:** 10.1016/j.ajoc.2022.101389

**Published:** 2022-02-03

**Authors:** Yoshikazu Ito, Takafumi Nakano, Mutsuko Ohara, Eisuke Shimizu, Yoko Ogawa, Kazuno Negishi

**Affiliations:** a Department of Ophthalmology, Keio University School of Medicine, Tokyo, Japan; b Ito Eye Clinic, Ibaraki, Japan; c Department of Ophthalmology, Tachikawa Hospital, Tokyo, Japan; d Division of Biological Science, Graduate School of Science, Kyoto University, Kyoto, Japan

**Keywords:** Leech, Worm, Ocular infestation, Parasite, Praobdellidae, *Myxobdella sinanensis*

## Abstract

**Purpose:**

The case of ocular infestation by a leech is rare. We reported that *Myxobdella sinanensis* infests conjunctiva.

**Observations:**

A 5-year-old girl presented with blood clots in the inner corner of the left eye, and a history bloody eye discharge and bloody tears for 5 days. She was prescribed 0.5% levofloxacin ophthalmic drops for conjunctival damage. However, her parent watched a worm moving in her conjunctiva while taking a bath. She presented again the same day, and a worm was found in the left eye of the lower conjunctival fornix and was adsorbed to the inner corner. We removed a worm under eye drop anesthesia, the next day the patient had no symptoms. We captured the worm, and it was identified morphologically and genetically as *Myxobdella sinanensis.* This was the first case reported of *Myxobdella sinanensis* be infestation in a human.

**Conclusions and Importance:**

The ecological trait of *Myxobdella sinanensis* still did not remain clear, so this case report was helpful to find out a life cycle of *Myxobdella sinanensis*. As the outdoor population continues to increase, the cases of human parasites such as leech are expected to increase. When a patient with bloody eye discharge and bloody tears presents, we should carefully examine the conjunctiva and ocular surfaces, and interview recent history of exposure to stream water.

## 1 Introduction

Pathogens of ocular infections include viruses, bacteria, fungi, protozoans, helminths and ectoparasites.^1^ Ectoparasites which cause ocular infestation are myasis, phthiriasis palpebrum, ticks, and leeches etc, however the case of ocular leech infestation is rare, and few cases were reported.^2^, ^3^, ^4^, ^5^, ^6^, ^7^, ^8^, ^9^

The leech is a parasitic organism, which belongs to the phylum Annelida, with an elongated body and suckers that attach to the body surface to suck body fluids or blood. They feed on crustaceans, fishes, birds, and mammals. To date, approximately 70 species have been recorded from Japan, however most of them are proboscidate or macrophagous species.^10^ Only six jawed blood-sucking species, which can infest vertebrates, are known to inhabit Japanese Archipelago: namely, *Hirudo nipponia* (Hirudinidae); *Haemadipsa japonica*, *Haemadipsa rjukjuana*, *Chtonobdella palmyrae* (Haemadipsidae); *Dinobdella ferox* and *Myxobdella sinanensis* (Praobdellidae).^10^, ^11^, ^12^, ^13^

These six species have been reported to infest ocular and nasal mucosa of vertebrates. To date, three species among them, *Hi. nipponia*, *Ha*. *japonica*, and *D. ferox*, were reported as a parasite of the human eye in Japan, but their precise identity still remains unclarified due to the lack of sufficient references of leech identification for the medical profession.^2^^,^^3^

We have recently encountered a case in which a leech infested a human eye. We provide herein its detailed pathological condition, as well as the authentic identity of the leech individual.

## 2 Case report

**Fig. 1:**
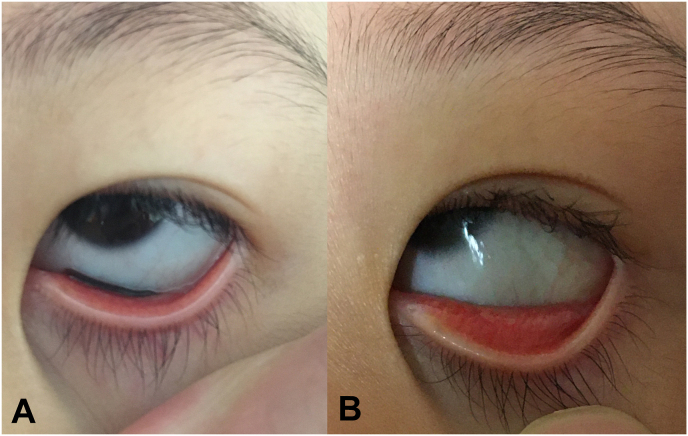
Ophthalmological examinations images of a left eye of a 5-year-old girl who had climbed a stream at mountain 6 days ago. A leech was observed on the lower conjunctival fornix and it suck the inner corner of conjunctiva (A). The patient's ocular finding after removal of a worm body showed almost normal (B).

A 5-year-old girl living in Ibaraki Prefecture, Japan, visited Ito Eye Clinic on July 30, 2020, because she began to have bloody eye discharge and bloody tears every morning after climbing a stream on July 25, 2020, at Mt. Tsukuba. At the time of the first visit, blood clots were observed in the inner corner of the left eye, and 0.5% levofloxacin ophthalmic drops were prescribed for conjunctival damage. After returning home, while the child was taking a bath, a family member complained of seeing moving insect bodies on the conjunctiva, and the patient presented again on the same day. At the time of re-examination, a parasite extending to a length of about 10 mm was found in the left eye of the lower conjunctival fornix so as to be adsorbed to the inner conjunctiva (Fig. 1). 0.4% Oxybuprocaine Hydrochloride drops was applied and the worm was removed by rubbing with a cotton swab using an eyelid opener. After the removal of the worm body, the conjunctiva of the patient was not hyperemic, and there was only a small amount of pale bloody exudate. When patient presented on July 31, she had no symptom and her ocular findings were normal.

## 3 Material and methods

**Fig. 2:**
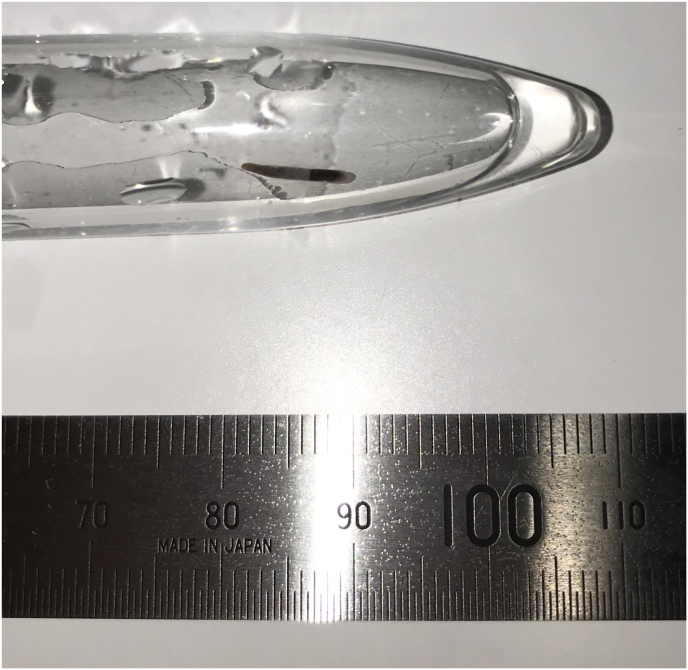
We captured a worm of about 10 mm length and we kept it alive in a plastic case with tap water.

The worm body was captured, stored in tap water, and transported alive by cool delivery to Kyoto University on August 24 for analysis (Fig. 2, Video. 1). The leech was preserved in absolute ethanol, and deposited in the Zoological Collection with the collection number KUZ Z3814 (body length, 8.4 mm). Due to the insufficient preservation of the specimen, we could only determine a short fragment (INSD accession number LC635508; 221 bp) of its mitochondrial cytochrome *c* oxidase subunit I (cox1) sequence. However, the leech has been unquestionably identified as a juvenile of ‘Praobdellidae gen. sp.’ (cf. Myxobdella sinanensis) given the fact that the present cox1 sequence was completely identical with the known cox1 sequence (INSD accession number LC192132; 1267 bp) obtained from an adult individual collected in Mt. Tsukuba.^14^^,^^15^

Supplementary video related to this article can be found at https://doi.org/10.1016/j.ajoc.2022.101389

The following is the supplementary data related to this article:

**Video. 1** A movie of a captured worm in a plastic case. It absorbed the surface of a plastic case and it repeated the extension and the shrinking.

## 4 Discussion

Ocular leech infestation is one of the differential diagnoses of conjunctival foreign body, after outdoor activity, especially in lakes or streams. It is reported that ocular parasitic cases of leeches may be misdiagnosed as conjunctivitis, if the color of the leech was brownish, conjunctival pigmented nevus or ocular trauma with iris prolapse because the patients don't complain strong eye discomfort.^4^, ^5^, ^6^ In this case, when the patient had awareness of the worm body wandering into the eye, it was easy to be considered as ocular leech infestation. However, at her first visit, because blood clots were attached to the worm and the worm body was supposed to be shrunken, it was difficult to find. When the possibility of ocular parasitism should be considered, the history of outdoor activities should be investigated.

It also has been reported that when we remove a leech, we should use anesthetic eye drops.^2^^,^^3^^,^^7^, ^8^, ^9^ Because the leech is strongly adsorbed to the conjunctiva and it is difficult to directly remove with forceps. Then we should use eye drop anesthesia to weaken its power of sucking to the ocular surface and can remove it safely. In this case, we chose 0.4% Oxybuprocaine Hydrochloride drops, and we could remove the leech easily by a cotton swab. Then patient didnot feel eye pain and her conjunctiva was almost normal without conjunctival hemorrhage.

**Fig. 3:**
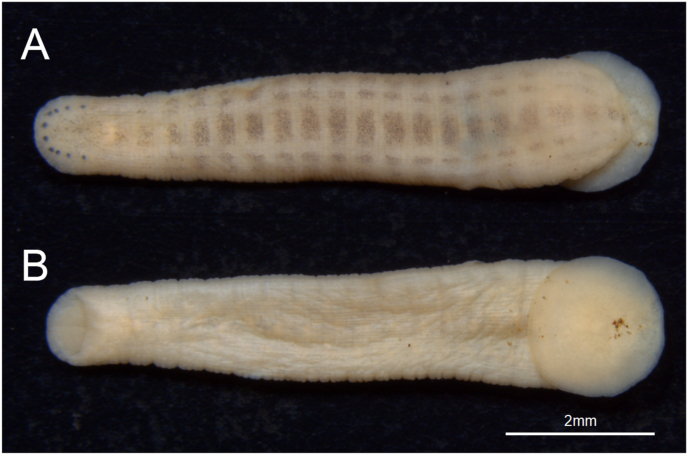
The preserved specimen (KUZ Z3814) of a juvenile *Myxobdella sinanensis.* Dorsal view showing its eyes in give pairs in parabolic arc (A). Ventral view showing its caudal sucker width slightly wider than the maximum body width (B).

We have encountered a case of human conjunctival parasitism with a juvenile leech, *Myxobdella sinanensis*, which has not been reported in humans. The ecological trait of *Myxobdella sinanensis* still did not remain clear, nonetheless, this species is known to inhabit mountain streams in Honshu island of Japan.^14^, ^15^, ^16^ The juvenile of *Myxobdella sinanensis* can be identified by the following three features; 1) eyes in five pairs in parabolic arc; 2) caudal sucker width almost similar to, or wider than maximum body width; 3) body surface whitish, with brownish markings forming 5 longitudinal broken stripes, median stripe widest (Fig. 3).

Praobdellid leeches comprise mucous-membrane specific leeches as well as species that prey upon freshwater crabs.^17^ However, only *Myxobdella sinanensis* is assumed to infest both vertebrates (mucous-membrane of the bird, *Ixobrychus eurhythmus*) and invertebrates (the freshwater crab, *Geothelphusa dehanni*).^12^^,^^14^ Therefore, the present case report can also support the possibility that this species seems to change its host/prey preference as it grows.

## 5 Conclusions

We reported the case of ocular infestation by a juvenile leech, and the leech was identified morphologically and genetically as *Myxobdella sinanensis.* This was the first case in which *Myxobdella sinanensis* infested a human, and we could reveal the ecological trait of *Myxobdella sinanensis*.

Ocular infestation by a leech could be misdiagnosed or missed. When a patient with bloody eye discharge and bloody tears presents, we should carefully examine the conjunctiva and ocular surfaces, and ask about recent exposure to mountain streams.

## Patient consent

The patient and the parents consented to publication of the case in orally. This report does not contain any personal information that could lead to the identification of the patient.

## Funding

The research leading to this case report was supported by 10.13039/501100001691JSPS KAKENHI Grant Number JP18K14780: TN.

## Authorships

All authors attest that they meet the current ICMJE criteria for Authorships.

YI, TN, and YO were major contributors in writing the manuscript. MO mainly treated and followed patient. Material preparation and analysis were performed by TN. All authors take responsibility for the integrity of the work as a whole and approved the final manuscript to be published.

## Declaration of competing interest

The authors declare that they have no conflict of interest.
